# Varicose Veins

**Published:** 1864-05

**Authors:** 


### VARICOSE VEINS.

Mr. Skey remarked (*Lancet*, Jan. 2, 1864), that the treatment of varicose veins involves two objects: "1st, the increase of power to these organs; and 2d, the turning the current of the venous circulation into healthier channels. The first is effected by the liberal administration of nutrio-stimulants. The second object has tested the inventive faculties of many surgeons. I leave it to others to commend the various schemes adopted by them. I discountenance, from long observation of its incompetency to cure, the employment of the needle, whether through the vein or under it, single or double. It has these objections: 1st, it is not unattended with danger; and 2d, it fails to obliterate the vein, except at the point of its application, mainly because the applications cannot be safely made in numbers proportionate to that of the veins affected. I have at present, in St. Bartholomew's Hospital, a woman, under treatment for varicose veins in the leg, whose limb was jeopardized by the employment of the needle a year ago. A long illness, with severe inflammation and extensive abscesses followed. The same limb is again under treatment for the original disease. There is no danger in making any number of small eschars on the most projecting surfaces of varicose veins, if made with an escharotic composed of two-fifths of pure potash and three-fifths of powdered lime. This powder well combined, is made into a paste with alcohol. Whether other escharotics are dangerous in their operation on veins I do not stop to inquire; I only know that the Vienna paste, combined as I have above described it, is not. These eschars may be made in any number proportionate to the extent of the disease. I have treated perhaps 250 cases in the course of the last ten years, and I continue to treat them, by the same means. The paste is applied over the most projecting part of the vein in the following manner; through a series of about four layers of adhesive plaster, a circle is cut of the size of a threepenny-piece or smaller. The influence of the escharotic extends through the vein; and it is curious to observe that from the hour of its application the entire vein appears to be obliterated, and is undetectable to the finger on pressure. From ten to twenty-five eschars may be applied between the ankle and the knee. Twenty minutes suffice for the full operation of the escharotic, and an average of one month for the cure. In very weak constitutions the ulcers will heal very slowly, unless well-directed efforts be made to give force to the general system."—*Am. Jour. of Med. Sciences.*

